# Automatic filtering of outliers in RR intervals before analysis of heart rate variability in Holter recordings: a comparison with carefully edited data

**DOI:** 10.1186/1475-925X-11-2

**Published:** 2012-01-11

**Authors:** Marcus Karlsson, Rolf Hörnsten, Annika Rydberg, Urban Wiklund

**Affiliations:** 1 Department of Radiation Sciences, Biomedical Engineering, and Centre of Biomedical Engineering and Physics, Umeå University, Umeå, Sweden; 2 Department of Surgical and Perioperative Science, Clinical Physiology and Heart Centre, Umeå University, Umeå, Sweden; 3 Department of Clinical Sciences, Pediatrics, Section of Pediatric Cardiology, Umeå University, Umeå, Sweden

## Abstract

**Background:**

Undetected arrhythmic beats seriously affect the power spectrum of the heart rate variability (HRV). Therefore, the series of RR intervals are normally carefully edited before HRV is analysed, but this is a time consuming procedure when 24-hours recordings are analysed. Alternatively, different methods can be used for automatic removal of arrhythmic beats and artefacts. This study compared common frequency domain indices of HRV when determined from manually edited and automatically filtered RR intervals.

**Methods and Results:**

Twenty-four hours Holter recordings were available from 140 healthy subjects of age 1-75 years. An experienced technician carefully edited all recordings. Automatic filtering was performed using a recursive procedure where RR intervals were removed if they differed from the mean of the surrounding RR intervals with more than a predetermined limit (ranging from 10% to 50%). The filtering algorithm was evaluated by replacing 1% of the beats with synthesised ectopic beats. Power spectral analysis was performed before and after filtering of both the original edited data and the noisy data set. The results from the analysis using the noisy data were used to define an age-based filtering threshold. The age-based filtration was evaluated with completely unedited data, generated by removing all annotations from the series of RR intervals, and then comparing the resulting HRV indices with those obtained using edited data. The results showed equivalent results after age-based filtration of both the edited and unedited data sets, where the differences in HRV indices obtained by different preprocessing methods were small compared to the mean values within each age group.

**Conclusions:**

The study showed that it might not be necessary to perform the time-consuming careful editing of all detected heartbeats before HRV is analysed in Holter recordings.

In most subjects, it is sufficient to perform the regular editing needed for valid arrhythmia analyses, and then remove undetected ectopic beats and artefacts by age-based filtration of the series of RR intervals, particularly in subjects older than 30 years.

## Background

Power spectrum analysis of the heart rate variability (HRV) is frequently used to study the cardiac autonomic modulation in patients with different diseases, as well as in healthy subjects during different experimental conditions [1]. Although the beat-to-beat fluctuations in heart rate most often are associated with cardiac autonomic modulation of the sinus node, the fluctuations are also caused by changes in heart rate due to cardiac rhythm and conduction disturbances. Arrhythmic beats and artefacts that are undetected during the ECG signal preprocessing seriously affect the power spectrum of the HRV [2-5]. Therefore is the series of RR intervals normally carefully edited before HRV is analysed. Editing is time-consuming, in particular when data from 24-hour ECG recordings (Holter) are analysed. Therefore, modern Holter systems include different methods to automatically remove undetected arrhythmic beats and artefacts [6-8]. However, which approach is preferable - editing or automatic filtering of RR intervals? In this study, we examined the effect of automatic filtering on frequency domain HRV indices. We hypothesized that there would be no clinically relevant differences in the calculated HRV parameters, when calculated from automatically filtered RR intervals as compared to manually edited RR intervals.

## Methods

### Study group

The study is based on data from 140 subjects (73 male and 67 female) that were retrieved from a local database of previously performed Holter recordings in healthy adult and young subjects. Since HRV varies with age, the subjects were divided into four groups: 1) children of age 1-14 years (mean age 9 years, n = 25); 2) young subjects of age 15-24 years (mean age 19 years, n = 32); 3) middle-aged subjects of age 25-49 years (mean age 38 years, n = 40); 4) old subjects of age 50-75 years (mean age 62 years, n = 43). All subjects were volunteers and had normal resting ECG:s. None of the subjects were taking any medicines that interfere with cardiovascular regulation.

### Holter-ECG recordings

All subjects underwent standard 24-hour ambulatory electrocardiographic monitoring during daily activity, using a standard recorder unit (Braemer DL 700, Braemer inc. Burnsville, MN, USA). ECG data were digitized with a sampling rate of 128 Hz. The ECG recordings were analysed using a PC-based Holter system (Aspect Holter System, GE Healthcare, Borlänge, Sweden). All recordings were evaluated using regular procedures for analysis of standard 24-hour ambulatory ECG recordings (Holter), which included assessment of underlying rhythm, cardiac conduction disturbances and the presence and frequency of arrhythmic beats.

During the Holter analysis all heartbeats were annotated as: normal, supraventricular extrasystolic beats, ventricular extrasystolic beats, extended RR-intervals, or beats of uncertain origin. The same investigator carefully examined all recordings and confirmed the classification of heartbeats. The editing time varied from one hour up to three hours, depending on the quality of the ECG-signal. The RR intervals and annotation of heartbeats were exported from the software for further analyses using Matlab (MathWorks Inc, Natick, Ma, USA).

### Data sets

Three different data sets were generated based on the carefully edited series of RR-intervals and the corresponding annotations of each beat:

*• Edited data set*, consisting of all RR intervals that were associated with two consecutive normal sinus beats, excluding the first RR interval after a non-sinus beat, e.g., the first sinus beat after an ectopic beat that corresponds to the compensatory pause.

*• Noisy data set*, constructed by inserting synthesised ectopic beats in the edited data set, as described below.

*• Unedited data set*, constructed by removing all annotations of heart beats, which corresponded to the worst case of completely unedited data. Thus, the unedited data set included RR intervals resulting from all types of beats, such as normal beats, ectopic beats and artefacts.

### Synthesized ectopic beats

To investigate the performance of the filtering algorithm regarding its ability to remove ectopic beats that were undetected during the editing, we replaced 1% of the normal beats in the edited data set with synthesised beats, imitating premature supraventricular ectopic beats [7]. RR intervals corresponding to ectopic beats were constructed to be between 30% and 100% of the average of the four preceding RR intervals using uniformly distributed random numbers. The RR interval following the ectopic beat was adjusted so that the average RR interval in the recording was unchanged. The number of synthesised ectopic beats was selected based on the average number of non-sinus beats in the recordings, which was up to 1%. The random generator was also used to randomly select where the ectopic beats were inserted, with the constraint that there had to be at least one normal RR interval between two ectopic beats. The generator was initiated with the same seed in order to generate the same sequence of where the ectopic beats should be inserted in all files.

### Automatic filtering of RR-interval data

All data sets were filtered using a recursive filtering procedure [8,9]. In each iteration, we removed all RR intervals that differed more than a predetermined limit from the mean of the preceding and following RR intervals. The majority of outliers were removed in the first step, but to remove sequences with several successive outliers, such as during supraventricular tachycardia, the filtering was repeated on the remaining series of RR intervals until no values were removed. However, the maximum number of iterations was set to 20 to avoid that all values from sequences with very rapid changes in heart rate were removed. The *noisy data set *was used to evaluate the filtering algorithm, using five different thresholds: 10, 20, 30, 40 and 50%, respectively.

### Age-dependent filtering

Based on findings during the evaluation of the filtering algorithm using synthesised ectopic beats, we designed an age-dependent threshold, chosen to be linearly increasing from 20% at age 1 year up to 40% at age 15 years, and then linearly decreasing down to 20% at age 75 years. The effect of filtering using the age-based threshold was evaluated by comparing HRV indices before and after filtering of the *edited data set *and *the unedited data set*, respectively.

### Frequency domain analysis

HRV was quantified before and after filtering of each data set by power spectrum analysis. The irregularly sampled series of RR intervals were converted to a time series by cubic spline interpolation, followed by resampling at two Hz. Data from the 24-hour recording were divided into 5-minute segments. The mean RR interval was removed, and data was smoothed using a Bartlett-window before power spectra were estimated according to the Welch method, using the fast Fourier transformation (FFT) calculated in 4096 points. The preprocessing of data also included replacement of removed data by interpolation, but 5-minute segments with more than 4% interpolated data were discarded. The percentage of segments remaining for frequency domain analysis was used as indicator of the recording's quality, i.e., the presence of arrhythmia and artefacts. In this study, we only included recordings with more than 70% used time when HRV was determined using the original edited data.

The following HRV indices were calculated as averages over the 24 hour recording: the total power in the frequency region 0.003-0.50 Hz (P_tot_); and the power of the very low frequency (P_VLF_, below 0.04 Hz), the low-frequency (P_LF_, 0.04-0.15 Hz) and the high-frequency (P_HF_, 0.15-0.50 Hz) components. The presence of a broadband pattern, i.e. a power spectrum without any distinct peaks, was used as an indicator of undetected non-sinus beats in the series of edited RR intervals as we previously suggested [9].

### Time domain analysis

Since different time domain indices are common in many HRV studies, we applied the algorithm for age-based filtration also on three such indices [1]. SDNN (standard deviation of normal RR intervals) was analysed as a marker of the long-term variability, and RMSSD (the square root of mean squared differences of successive normal RR intervals) and pNN50 (the number of successive normal RR intervals that differ more than 50 ms divided with the total number of normal RR intervals) as markers of the beat-to-beat variability.

### Statistical analyses

Frequency domain HRV indices were log-transformed (base 10) because of skewed distributions. Analysis of variance (ANOVA) for repeated measurements was used for comparison of the different methods for preprocessing of RR intervals in the four age groups.

The clinical relevance of the small differences found between preprocessing methods were investigated using the TOST test (two one-sided T-test) [10]. This test is an equivalence test where the H_0 _hypothesis is that the groups are similar, implying that the groups do not differ more than a threshold Ө, where the threshold may be based on heuristic information, such as estimated measurement errors. The H_1 _hypothesis is that the groups are not similar, implying that the difference between the groups is larger than Ө. The TOST test is equivalent to determine the 90% confidence intervals for the difference in a paired T-test, and reject the H_0 _hypothesis if the confidence interval is not completely contained within the interval (-Ө, Ө). We defined Ө = 5% as an acceptable difference in HRV parameters based on different data sets, corresponding to the asymmetric interval (Ө_L_, Ө_U _) = (-0.022, 0.0212) in log-transformed units. The level of statistical significance was defined as a two-tailed p value <0.05. Statistical analyses were preformed using SPSS (SPSS Inc, Chicago, IL, USA).

## Results

**Figure 1:**
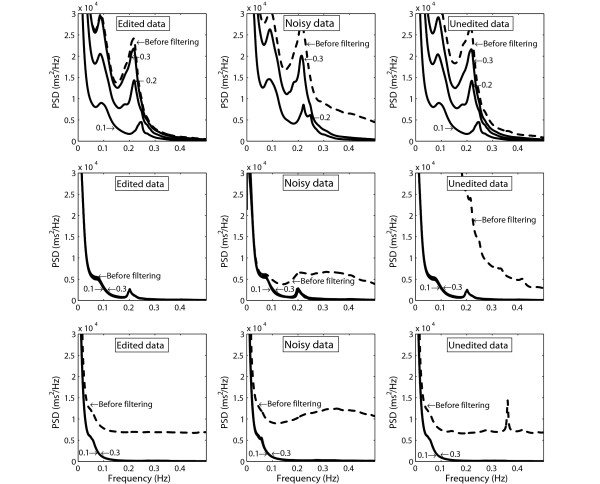
**Power spectra of HRV in three subjects before and after filtering of the different sets of RR intervals: edited data (left); noisy data (middle); and unedited data (right)**. Top: subject with high HRV. Middle: subject with relatively low HRV. Bottom: subject where frequent supraventricular ectopic beats were undetected during editing. Numbers in the figures indicate the threshold used for filtering.

Figure 1 shows examples of HRV power spectra before and after filtering of the three different data sets from three subjects. These subjects were chosen to show the effect of different thresholds in the automatic filter. The left panels shows power spectra determined from the series of edited RR intervals, the middle panels illustrate the effect of adding synthesised ectopic beats and then filtering, and the right panels shows power spectra for unedited data where all annotations had been removed before filtering was applied.

The upper panels show results from one subject with high HRV (age 20 years), where filtration with thresholds ≤20% resulted in markedly reduced HRV compared to the power spectrum for the edited data. HRV could not be determined after filtration of the noisy data with the threshold 10% because too many beats were removed. The middle panels show data from a subject with low HRV (age 70 years), where the effect of the synthesised ectopic beats was efficiently removed by filtering with all thresholds. This subject also showed a close resemblance between the power spectra for edited data and filtered unedited data. Finally, the lower panels show power spectra for a subject with low HRV (age 73 years) where frequent supraventricular ectopic beats were undetected during the editing, resulting in a broadband power spectrum before filtering.

### Filtering of synthesized ectopic beats

**Figure 2:**
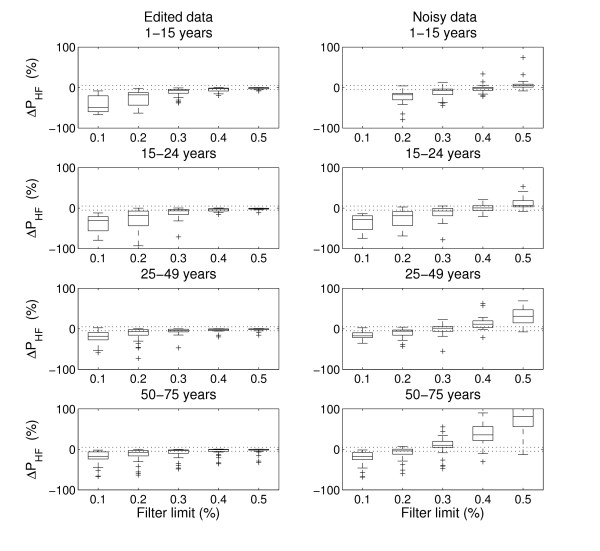
**Effect of filtering on high-frequency power for the edited data (left) and noisy data (right) for subjects in the different age-groups**. The box-and-whiskers plot show median, interquartile range, range and outliers for the change in high-frequency power after filtering. Horisontal lines indicate ±5% difference.

Figure 2 shows the difference between P_HF _calculated for the edited data and P_HF _after filtering of edited data (left panels) and noisy data (right panels), respectively. Filtration of edited data with the threshold 0.2 and lower resulted in a marked reduction of P_HF _in the two youngest age-groups, where a low value of the threshold removed many normal sinus beats resulting in an underestimated P_HF_. On the other hand, a threshold of approximately 0.2 was needed to reduce the marked increase in P_HF _due to the addition of noise for subjects in the two oldest age groups.

### Age-dependent filtering

**Figure 3:**
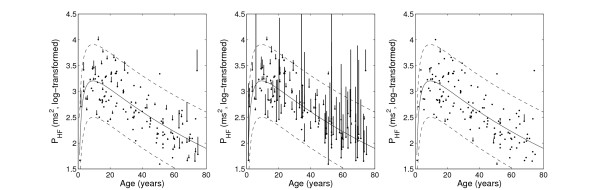
**High-frequency power based on edited and filtered data (dots) compared to: edited data (left); unedited data (middle); and unedited and filtered data (right)**. Vertical lines indicate the difference compared to edited and filtered data, but are only shown for subjects with >5% difference. Regression lines and 95% confidence intervals were determined from quadratic regression based on edited and filtered data and log-transformed values of age.

The overall age-dependency in HRV is illustrated in Figure 3. The figure also shows the comparison of P_HF _based on edited and filtered data with results based on the unfiltered unedited and filtered unedited data sets. Table 1 shows mean values of HRV parameters for each age-group before and after age-dependent filtering of the edited and unedited data sets, respectively. HRV calculated from edited data showed marginally higher values before filtering than after filtering: a result that was partly explained by the removal of a number of sinus beats in young subjects with relatively high HRV, but also by findings of broadband power spectra indicating the presence of undetected non-sinus beats in the series of edited data which was found in 6 subjects, all of age >59 years.

**Table 1:** Group averages of HRV for edited and unedited data, respectively, when calculated before and after filtering with the age-based threshold.

	*Before filtering*		*After filtering*	
	***Edited data***	***Unedited data***	***Edited data***	***Unedited data***

**P_tot _(ms^2^, log) (Age-group: p < 0.001, SS = 15.5; Method: p < 0.001, SS = 0.1)**				

1-14 years (N = 25)	3.71 (0.33)	4.14 (0.86)	3.68 (0.33)	3.68 (0.33)
15-24 years (N = 32)	3.90 (0.23)	4.09 (0.50)	3.90 (0.23)	3.88 (0.23)
25-49 years (N = 40)	3.64 (0.23)	3.89 (0.83)	3.64 (0.23)	3.63 (0.23)
50-75 years (N = 43)	3.40 (0.28)	3.73 (0.62)	3.35 (0.27)	3.36 (0.27)
**P_VLF _(ms^2^, log) (Age-group: p < 0.001, SS = 10.0; Method: p < 0.001, SS = 0.1)**				
1-14 years (N = 25)	3.36 (0.31)	3.82 (0.97)	3.35 (0.31)	3.36 (0.31)
15-24 years (N = 32)	3.63 (0.22)	3.81 (0.55)	3.63 (0.22)	3.61 (0.22)
25-49 years (N = 40)	3.38 (0.21)	3.60 (0.88)	3.38 (0.21)	3.35 (0.21)
50-75 years (N = 43)	3.21 (0.25)	3.43 (0.61)	3.16 (0.26)	3.17 (0.25)
**P_LF _(ms^2^, log) (Age-group: p < 0.001, SS = 19.4; Method: p < 0.001, SS = 0.004)**				
1-14 years (N = 25)	3.11 (0.29)	3.35 (0.56)	3.10 (0.30)	3.10 (0.31)
15-24 years (N = 32)	3.34 (0.26)	3.55 (0.41)	3.34 (0.26)	3.34 (0.26)
25-49 years (N = 40))	3.14 (0.26)	3.31 (0.52)	3.14 (0.26)	3.14 (0.26)
50-75 years (N = 43)	2.78 (0.34)	3.18 (0.63)	2.76 (0.32)	2.76 (0.32)
**P_HF _(ms^2^, log) (Age-group: p < 0.001, SS = 57.8; Method: p < 0.001, SS = 0.12)**				
1-14 years (N = 25)	3.12 (0.47)	3.31 (0.49)	3.06 (0.45)	3.05 (0.45)
15-24 years (N = 32)	3.10 (0.36)	3.26 (0.38)	3.10 (0.36)	3.08 (0.35)
25-49 years (N = 40))	2.68 (0.38)	2.92 (0.53)	2.68 (0.38)	2.67 (0.36)
50-75 years (N = 43)	2.26 (0.41)	2.86 (0.60)	2.19 (0.38)	2.20 (0.38)

Data are given as mean (SD).

P-values and sum of squares (SS) were derived from repeated measures ANOVA with age-group (df = 3) and method (df = 2) as factors. The unedited and unfiltered data were not included in the ANOVA.

The ANOVA showed statistically significant differences between the different methods (Table 1). However, note that the differences between the mean values obtained by each method were small, as compared to the differences between the mean values within each age-group. The equivalence test presented for the frequency domain indices with most marked differences in Table 2 showed that filtering of both edited and unedited data resulted in equivalent values of HRV parameters.

**Table 2:** Equivalence test of preprocessing methods.

	***a) Edited vs*** ***b) Edited and filtered***	***a) Edited vs*** ***c) Unedited and filtered***	***b) Edited and filtered vs*** ***c) Unedited and filtered***
ΔP_tot _(ms^2^, log)			
Subjects 1-14 years (N = 25)	(0.014, 0.042)*	(0.012, 0.039)*	(-0.006, 0.001)
Subjects 15-24 years (N = 32)	(0.020, 0.030)*	(0.014, 0.025)*	(-0.009, -0.002)
Subjects 25-49 years (N = 40)	(0.017, 0.026)*	(0.008, 0.019)	(-0.012, -0.004)
Subjects 50-75 years (N = 43)	(0.035, 0.067)*	(0.026, 0.059)*	(-0.012, -0.005)
**ΔP_HF _(ms^2^, log)**			
Subjects 1-14 years (N = 25)	(0.036, 0.085)*	(0.041, 0.089)*	(-0.001, 0.011)
Subjects 15-24 years (N = 32)	(0.015, 0.030)*	(0.007, 0.026)*	(-0.011, -0.001)
Subjects 25-49 years (N = 40)	(0.011, 0.027)*	(0.002, 0.021)	(-0.013, -0.003)
Subjects 50-75 years (N = 43)	(0.042, 0.096)*	(0.035, 0.085)*	(-0.014, -0.003)

Data are given as 90% confidence interval for difference. * p < 0.05 in TOST test using ±5% as acceptable difference, corresponding to (Ө_L_, Ө_U _) = (-0.022, 0.0212) in log-transformed units.

Summary of differences in HRV indices when calculated from differently pre-processed R-R intervals: a) edited data, b) edited and filtered data, and c) unedited and filtered data. Filtering of RR-intervals was performed using the age-based threshold.

Table 3 shows the corresponding differences between methods and age-groups for the time domain indices.

**Table 3:** Group averages of HRV (time domain) for edited and unedited data, respectively, when calculated before and after filtering with the age-based threshold.

	*Before filtering*		*After filtering*	
	***Edited data***	***Unedited data***	***Edited data***	***Unedited data***

**SDNN (ms) (Age-group: p < 0.001, SS = 110402; Method: p = 0.81, SS = 1202)**				

Subjects 1-14 years (N = 25)	157 (40.8)	253 (210)	155 (40.8)	156 (40.9)
Subjects 15-24 years (N = 32)	197 (50.1)	213 (86.3)	197 (50.0)	198 (49.8)
Subjects 25-49 years (N = 40)	162 (34.5)	266 (435)	161 (34.2)	162 (34.4)
Subjects 50-75 years (N = 43)	167 (97.9)	190 (113)	153 (46.7)	154 (46.7)
**RMSSD (ms) (Age-group: p < 0.001, SS = 88214; Method: p = 0.002, SS = 1299)**				
Subjects 1-14 years (N = 25)	64.8 (33.9)	133 (198)	60.0 (30.4)	60.1 (30.3)
Subjects 15-24 years (N = 32)	66.6 (29.8)	103 (126)	64.3 (27.9)	64.9 (27.8)
Subjects 25-49 years (N = 40)	42.1 (18.5)	118 (257)	40.5 (16.9)	41.1 (16.7)
Subjects 50-75 years (N = 43)	35.0 (30.0)	84.8 (93.2)	27.6 (15.3)	28.1 (15.3)
**pNN50(%) (Age-group: p < 0.001, SS = 27941; Method: p < 0.001, SS = 9.4)**				
Subjects 1-14 years (N = 25)	26.7 (13.8)	26.9 (13.7)	26.1 (13.4)	26.0 (13.3)
Subjects 15-24 years (N = 32)	27.3 (12.9)	27.6 (12.9)	27.1 (12.7)	27.2 (12.7)
Subjects 25-49 years (N = 40)	15.7 (9.8)	16.3 (9.7)	15.6 (9.7)	15.7 (9.7)
Subjects 50-75 years (N = 43)	7.9 (10.3)	8.9 (10.2)	7.4 (9.3)	7.5 (9.2)

See Table 1 for details.

Data are given as mean (SD).

## Discussion

This study compared common frequency domain indices of HRV calculated from carefully edited and automatically filtered RR intervals, respectively. Although the results showed statistically significant differences between HRV indices obtained by preprocessing methods, the magnitude of the differences were small compared to the mean values of each parameter. By defining ±5% as a clinically acceptable difference, equivalent results were found when filtering was applied to both the edited and the "unedited" data set. Both statistically significant and clinically relevant differences were found between subjects in different age groups, where HRV increased from birth to approximately 15 years and then decreased, as expected from many previous studies, e.g., [11,12].

Because of the marked differences in HRV between subjects of different age, the study introduced an age-based threshold for filtering of RR intervals, as a trade-off between the risks of removing normal sinus beats, particularly in subjects with high HRV, and the susceptibility to falsely detected beats, such as spikes due to noise and undetected supraventricular ectopic beats. The analysis of the noisy data set also pointed to the necessity of using age-adaptive algorithms for filtering of RR intervals.

Careful editing of Holter recordings is time consuming, taking up to several hours in 24-hour recordings. Today there are Holter recorders that can record data up to 7 days, and editing of all beats in such long recordings is an impossible task. No matter how carefully the editing is performed, a number of supraventricular beats will probably always be undetected during the editing of Holter recordings, as also pointed out in previous studies [6,8,9], [13]. Another source of errors in RR intervals is detection errors, either in the automatic analysis or due to manual insertion of missed beats. If automatic filtering of RR intervals is performed, our results show that careful editing may not be necessary before HRV is analysed in Holter recordings. However, we recommend that automatic filtering always is performed to remove undetected non-sinus beats, otherwise the results can be misleading since the HRV parameters may be markedly overestimated, as in the subject shown in Figure 1 (last example).

The results in this study do not only apply to healthy subjects. In a previous study, we applied the algorithm for filtering of RR intervals in patients with the disease familial amyloidotic polyneuropathy where both reduced HRV and cardiac arrhythmia are common findings [9]. These patients often present recordings with extremely low HRV as well as episodes with "falsely" increased HRV because of subtle atrial arrhythmias. This subtle atrial arrhythmia is often difficult to detect during the normal editing of Holter recordings, but results in markedly increased HRV [9,13]. However, the study showed that automatic filtering of RR intervals was efficient to reduce the influence that subtle atrial arrhythmia had on HRV. Since then, we are always applying automatic filtration to remove undetected ectopic beats, after performing the normal editing that is needed for reliable arrhythmia analyses of Holter recordings.

As shown in Figure 3, a notable difference in HRV was found in several subjects before and after filtering of carefully edited data. This can partly be explained by undetected non-sinus beats, and partly by the fact that the filtering probably also removed a number of sinus beats in subjects with high HRV (these subjects were well above the regression line also after filtering). Therefore, in subjects where a large proportion of beats are removed after filtering, it is always necessary to re-investigate the unfiltered data to find the reason why so many beats were removed, e.g., by inspection of the beat-to-beat fluctuations in the heart rate tachogram at an appropriate scale to identify outlier beats. This analysis can also be performed by inspection of the so called Poincaré plots, a tool that we and others use in all HRV analyses based on Holter-ECG:s [6,14]. The pattern in the Poincaré plot often identifies subjects that have high HRV but very irregular or "erratic" heart rhythms [6], where many beats probably will be considered as outliers and removed by the algorithm used for filtering. Thus, automatic filtering of RR intervals could be used as a first step for *identifying *subjects with complex heart rhythms, but for those subjects the original unfiltered data should be used in further analyses, bearing in mind that the frequency domain analysis must be interpreted with caution.

To evaluate how the filter performed with noisy signals we generated artificial ectopic beats by changing the time instant for a number of beats that were classified as normal. Already at a level of 1% ectopic beats there was a significant difference between HRV parameters for the edited and noisy data sets. This is in concordance with the study by Stork *et al *who showed that even a small number of ectopic beats affect the analysis of HRV both in the time and frequency domain [7].

A novel approach in our study was the use of an age-dependent threshold in the algorithm for automatic filtering of RR intervals. This was motivated since the sensitivity to noise were less marked in young subjects with high HRV, as compared to the marked influence of ectopic beats in old subjects with lower HRV, as illustrated in Figure 1. Thus, when filtering of data from subjects in the oldest age-group, a relatively low value of the filtering threshold should be used to remove the ectopic beats. If the same filter limit would be used in old subjects as in young subjects, too many of the normal beats would be removed which would result in too low values of the HRV parameters. The evaluation of the filtering algorithm also showed that the power of the high-frequency component of HRV is the parameter that was most sensitive to undetected ectopic beats, as expected since the added noise consisted of rapid beat-to-beat changes in the RR intervals. Thus, when using a filtering algorithm based on age, the choice of threshold is a trade-off between the ability of removing ectopic beats and the risk for removing normal sinus beats in subjects with pronounced respiratory sinus arrhythmia. Still, our overall aim is to remove all the ectopic beats to make sure that the HRV analysis is performed only on beats triggered from the sinus node. Whichterle used a filter limit of 10% to be sure that all the ectopic beats were removed before calculation of HRV in data from elderly patients. Stein found that on elderly a filter limit of 10% reduced the appearance of ectopic beats on Poincaré plots, but a level of 20% did not. Also note that elderly subjects have more ectopic beats and lower HRV than young subjects and must be filtered with a lower filter limit.

Finally, it is important to visually investigate the unfiltered series of RR intervals in all recordings where a large amount of beats have been removed after filtration. Why were the beats removed? Was it because of undetected ectopic beats, because of many artefacts in the recording, or were normal sinus beats removed in subjects with high HRV? This is especially important in patient groups where reduced HRV can be expected because of autonomic dysfunction. In this type of groups it may also be necessary to choose an even lower filter limit then the age-based threshold suggested in this study.

There are some limitations of the present study. The performance of automatic filtering was investigated using edited data from healthy subjects where synthesised noise was added. Even though the Holter recordings were edited more carefully than normally, a number of incorrectly annotated beats were found in some recordings. Moreover, the study only investigated the effect of spurious single ectopic beats. Therefore, the algorithm for automatic filtering was not evaluated using data containing sequences of successive synthesised ectopic beats.

## Conclusions

Our study showed that automatic filtering resulted in equivalent quality of HRV parameters as after careful editing, where the magnitude of the differences was small compared to the mean values of each parameter. We also defined a novel age-based threshold to increase the efficacy of automatic filtration since there were marked differences in HRV between subjects of different age.

Careful editing of all detected beats in a Holter recording may not be necessary before the analysis of HRV. Instead, it is probably sufficient to perform the normal editing that is needed for the regular arrhythmia analyses of Holter-ECGs. But then one should always apply automatic filtering to remove undetected non-sinus beats. Thus, automatic filtration simplifies the analysis of HRV in long-term recordings, decreases the risk that subjects have falsely increased HRV because of undetected subtle arrhythmias, and also helps to identify subjects with very irregular sinus rhythms.

## Abbreviations

**ANOVA**: analysis of variance; **ECG**: electrocardiogram; **HF**: high- frequency; **HRV**: heart rate variability; **LF**: low frequency; **TOST**: two one-sided T-test; **VLF **- very low frequency.

## Competing interests

The authors declare that they have no competing interests.

## Authors' contributions

All authors contribute in the design of the study and participated in the preparation and revision of the manuscript, thus all authors made substantial contributions to the scientific content. All authors read and approved the final manuscript. Specifically, MK and UW performed data analyses and had the main responsibility for writing of the manuscript; AR evaluated Holter recordings in young subjects; RH: edited all Holter recordings and preformed all arrhythmia analyses.
